# Functional and Seasonal Changes in the Structure of Microbiome Inhabiting Bottom Sediments of a Pond Intended for Ecological King Carp Farming

**DOI:** 10.3390/biology11060913

**Published:** 2022-06-14

**Authors:** Agnieszka Wolińska, Anna Kruczyńska, Jarosław Grządziel, Anna Gałązka, Anna Marzec-Grządziel, Klaudia Szałaj, Agnieszka Kuźniar

**Affiliations:** 1 Department of Biology and Biotechnology of Microorganisms, The John Paul II Catholic University of Lublin, 1 I Konstantynów Str., 20-708 Lublin, Poland; anna.kruczynska@kul.pl (A.K.); klaudia.szalaj1998@gmail.com (K.S.); agnieszka.kuzniar@kul.pl (A.K.); 2 Department of Agriculture Microbiology, Institute of Soil Science and Plant Cultivation, Czartoryskich 8 Str., 24-100 Puławy, Poland; jaroslaw.grzadziel@gmail.com (J.G.); agalazka@iung.pulawy.pl (A.G.); agrzadziel@iung.pulawy.pl (A.M.-G.)

**Keywords:** bottom sediments, biodiversity, CLPP, NGS, microbiome, seasonality

## Abstract

**Simple Summary:**

Bottom sediments are usually classified as extreme habitats for microorganisms. They are defined as matter deposited on the bottom of water bodies through the sedimentation process. The quality of sediments is extremely important for the good environmental status of water, because they are an integral part of the surface water environment. Microorganisms living in sediments are involved in biogeochemical transformations and play a fundamental role in maintaining water purity, decomposition of organic matter, and primary production. As a rule, studies on bottom sediments focus on monitoring their chemistry and pollution, while little is known about the structure of bacterial communities inhabiting this extreme environment. In this study, Next-Generation Sequencing (NGS) was combined with the Community-Level Physiological Profiling (CLPP) technique to obtain a holistic picture of bacterial biodiversity in the bottom sediments from Cardinal Pond intended for ecological king carp farming. It was evident that the bottom sediments of the studied pond were characterized by a rich microbiota composition, whose structure and activity depended on the season, and the most extensive modifications of the biodiversity and functionality of microorganisms were noted in summer.

**Abstract:**

The main goal of the study was to determine changes in the bacterial structure in bottom sediments occurring over the seasons of the year and to estimate microbial metabolic activity. Bottom sediments were collected four times in the year (spring, summer, autumn, and winter) from 10 different measurement points in Cardinal Pond (Ślesin, NW Poland). The Next-Generation Sequencing (MiSeq Illumina) and Community-Level Physiological Profiling techniques were used for identification of the bacterial diversity structure and bacterial metabolic and functional activities over the four seasons. It was evident that Proteobacteria, Acidobacteria, and Bacteroidetes were the dominant phyla, while representatives of Betaproteobacteria, Gammaproteobacteria, and Deltaproteobacteria predominated at the class level in the bottom sediments. An impact of the season on biodiversity and metabolic activity was revealed with the emphasis that the environmental conditions in summer modified the studied parameters most strongly. Carboxylic and acetic acids and carbohydrates were metabolized most frequently, whereas aerobic respiration I with the use of cytochrome C was the main pathway used by the microbiome of the studied bottom sediments.

## 1. Introduction

Aquatic microbiota is a term used to describe the vast abundance and diversity of microorganisms inhabiting water environments [1]. They are estimated to constitute about 60% of the total biomass and are considered as the most diverse group of organisms in the entire biosphere [1,2]. In the aquatic environment itself, e.g., in surface waters including the oceans, the total number of prokaryotic cells can reach up to 10^29^, which indicates their undeniable role in the formation and functioning of aquatic ecosystems [1,3].

Microbes perform a number of important ecosystem services in water. Their greatest function is the primary production of energy from carbon dioxide (CO_2_) [1] and supporting water ecosystems through their involvement in nutrient cycling, especially the nitrogen cycle [4]. Usually, high levels of organic carbon are deposited in marine (aquatic) sediments, from which a portion of methane (CH_4_) is produced [3,5]. Consequently, the ocean contributes to approx. 2% of the global atmospheric CH_4_ budget [5,6]. In such conditions, usually microbial consortia of anaerobic methanotrophic Archaea and sulfate-reducing bacteria [7] and/or Chloroflexi and Gammaproteobacteria [8], Proteobacteria, Elusimicrobia, and Actinobacteria [2] are identified. Cyanobacteria, Deferribacteres, and Thaumarchaeota are also abundant, although the taxonomic richness within these phyla is smaller [2] than in the phyla mentioned above. In shrimp ponds with different population densities, bacterial communities have been found to be dominated by Gammaproteobacteria, Alphaproteobacteria, Flavobacteriia, Bacilli, and Actinobacteria [9].

Bottom sediments are composed of sedimentary material deposited on the bottom of rivers and water bodies. Fish ponds are rich in dissolved organic matter due to intensive feeding and fecal waste [10]. Ponds accumulate bottom sediments after their basins are formed under the influence of the water regime (emptying and filling). These sediments are formed from biological debris originating from the ponds and their catchments, as well as soil particles and other non-biological materials that have entered the water body [10]. Cellulolytic bacteria, numerous anaerobic chemoautotrophs, and anaerobic microbiota typically thrive in bottom sediments [10]. In the Arctic sediments in Baffin Bay, Cramm, et al. [11] noted the presence of putative methane-oxidizing *Methyloprofundus*, sulfate-reducing *Desulfobulbaceae*, and sulfide-oxidizing *Sulfurovum*. Lee, et al. [12] identified the presence of Proteobacteria, followed by Chloroflexi, Bacteroidetes, Acidobacteria, and Firmicutes in sediments in the Yellow Sea. In the sediments originating from Laoshan Bay (China), the bacterial community was dominated (>10%) by the phyla Proteobacteria, Desulfobacterota, and Acidobacteria [13]. It was also emphasized that the biodiversity of urban ponds, expressed by species richness, appears to be generally lower than in rural ponds [14].

However, the knowledge of bacterial inhabitants of bottom sediments (especially in the aspect of Polish stock ponds) is still limited and needs to be constantly extended. This was an inspiration for conducting the study presented in this paper, where we monitored changes in the bacterial structure over the seasons of the year taking into account the fact that temperature is one of the most important environmental drivers with the greatest influence on the water microbiome [2,15]. What is more, it was suggested that ponds are highly diverse but understudied systems that could benefit from eDNA monitoring [15]. Usually, assessment of pond biodiversity is regarded as costly, time-consuming, and dependent on taxonomic expertise [13,16]. Nevertheless, a culture-independent technique was used in this study to identify the highest possible number of bacteria capable of living in the bottom sediments of a pond intended for organic king carp culture.

Next-Generation Sequencing (NGS) was combined with the Community-Level Physiological Profiling (CLPP) technique to obtain a holistic picture of bacterial biodiversity in bottom sediments. We hypothesized that both the diversity of bacteria and their metabolic activity depend on the season of the year. The main goal of the study, in addition to the precise identification of the taxonomic structure of bacteria inhabiting bottom sediments, was to find out in which season the microbiome of farm ponds will be the most active and diverse both structurally and metabolically.

## 2. Materials and Methods

### 2.1. Description of the Breeding Pond (Cardinal Pond in Ślesin)

**Figure 1:**
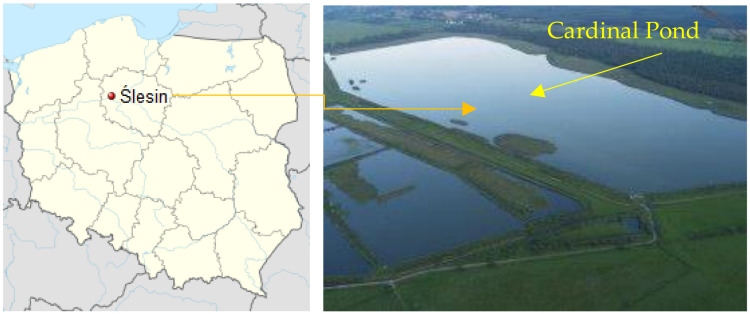
Location of the sampling area on the map of Poland and a view of Cardinal Pond.

Bottom sediments for the study were collected from Cardinal Pond located in Ślesin (53°09′52′′ N 17°42′16′′ E, Figure 1). Importantly, the Ślesin fish farm is located in an area that is protected under the European Natura 2000 network. Due to its proximity to the Noteć River and the Bydgoszcz Canal, the pond is well supplied with fresh and clean water, creating ideal conditions for king carp farming.

Moreover, Cardinal Pond is characterized by a fertile peat bottom, which is an excellent food base for fish. The studied reservoir has an area of 104 hectares (ha). It was built between 1934 and 1935, and its water surface area is 145.5 ha.

The tradition of carp farming in the Ślesin area is about 100 years old. Carp mature in ponds for 3 years, and their production is sustainable. The king carp feed on natural food that has formed in the pond ecosystem, hence the scientific aspect of the current study related to the assessment of biodiversity in bottom sediments is extremely important. In addition, the carp are fed with cereals from local crops. In spring (the beginning of April), the pond is stocked with about 80,000 carp crocs (in total about 20–25 tons; 3–4 carp/kg; one croc weighs about 300 g). Then, the fry are fed in summer and caught in autumn (November) (about 80 tons of carp are harvested), and the water is drained. During winter, the pond has no water, and bottom sediments are exposed to atmospheric conditions.

### 2.2. Bottom Sediment Sampling

**Figure 2:**
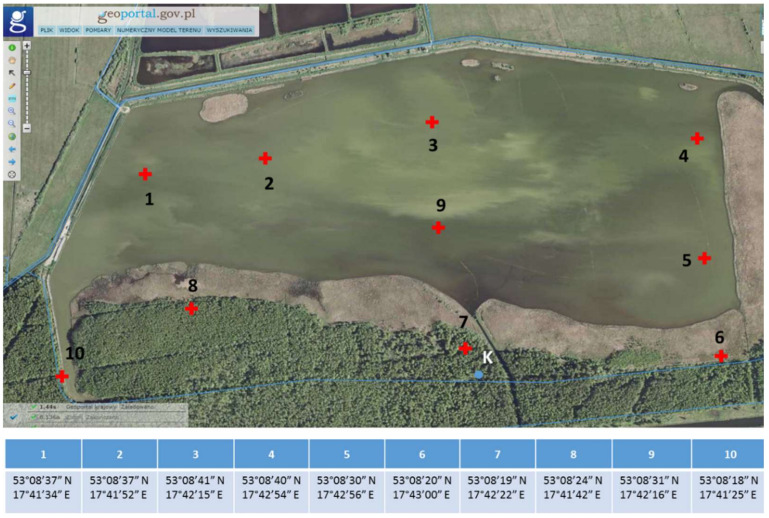
Location of the bottom sediment sampling points on Cardinal Pond.

Bottom sediment samples were collected from 10 different measurement points (as independent samples) placed in such a way as to obtain the most representative material for the study, taking into account the surface area of the pond (Figure 2).

The samples were collected 4 times in the following terms: spring (27 May 2020), summer (24 August 2020), autumn (26 October 2020), and winter (2 February 2021).

In the wintertime (December–March), after the fish are removed, the water is drained from Cardinal Pond and the sediments are exposed to the weather. Additionally, liming of the substrate is applied to maintain a pH of approximately 7. Therefore, in February 2021, sediment samples were collected from the natural reservoir.

Samples were collected from the assigned points based on GPS locations (Figure 2). Sediments were sampled from the surface layer of the tank bottom (approximately 1.2 m depth) using a vacuum-piston sampler (Royal Eijkelkamp, Giesbeek, The Netherlands). Approximately 1 L of sediments were collected at a time into labeled plastic boxes. To maintain the natural microbiological processes occurring in the sediments, the boxes were immediately sealed. The collected samples were stored at 4 °C until they were transported to the laboratory.

### 2.3. DNA Extraction and NGS Analysis

DNA was isolated using the DNeasy^®^ PowerLyzer^®^ PowerSoil^®^ kit (Qiagen, Hilden, Germany) as recommended by the manufacturer’s Quick-Start Protocol (Qiagen, Germantown, MD, USA). Approximately 0.300 g of sediment material was used for each of the 10 sampling points (in the relevant seasons), each in triplicate. Metabarcoding of 16S rRNA was performed within its V3–V4 region [17]. 341f and 785r primers were applied both for the amplification of the mentioned region and for the preparation of the library [17,18]. The PCR was carried out as described by Wolińska, et al. [17] with the use of Q5 Hot Start-High Fidelity 2X Master Mix (New England Biolabs INC., MA, USA). When a positive effect of PCR was obtained, triplicate DNA isolates of one sample were pooled, which was in agreement with the recommendation of Kuźniar, et al. [19]. Next-Generation Sequencing (NGS) was performed by Genomed S.A. on a MiSeq sequencer, paired-end (PE) technology, 2 × 300 nt, using Illumina kit v2.

### 2.4. Bioinformatic, Functional, and Statistical Analyses

Bioinformatic analyses of the sequences obtained were performed in R v4.1 using DADA2 v1.18 software [20]. The DECIPHER package v2.20 [20] based on the GreenGenes v13_8 reference database [21] was applied for classification of the sequences. The results are presented as relative abundance expressed as a percent of identified sequences at the phylum, class, and genus taxonomic levels.

The functional profile of the microbial communities was prepared in Linux using PICRUSt 2.0 software [22]. Predicted genes were compared with the Kyoto Encyclopedia of Genes and Genomes (KEGG) database. LEfSe analysis and RDA analysis were performed in R using the microeco package (v0.7.1) [23]. The graph was prepared in R using the heatmap package (v1.0.12) and the ggplot2 package (v3.3.5).

Statistical analyses were performed using STATISTICA 12.0 software. The significant differences in the KEGG analysis were compared via multiple *t*-tests.

All identified sequences are available under accession number PRJNA832534 (GenBank Database, NCBI: https://www.ncbi.nlm.nih.gov/bioproject/PRJNA832534 (accessed on 27 April 2022)).

### 2.5. Community-Level Physiological Profiling (CLPP)

The metabolic potential of the microbial communities was determined with the use of the Biolog EcoPlate containing 31 different carbon sources representing five groups (amines and amides, amino acids, carbohydrates, carboxylic acids, and polymers) [17]. Shortly, 1 g of bottom sediments was weighed, mixed with 99 mL of sterile 0.9% NaCl, and vortexed (30 min, 150 rpm, 25 °C). Then, all samples were cooled (30 min, 4 °C), transferred into each of the wells in the EcoPlate, and incubated in the dark (28 °C, 144 h). The experiment was carried out in three replications for each bottom sediment sample. The results were read every 24 h on the MicroStation ID system.

All results are expressed as a percentage of utilization of individual compounds divided into particular groups and are also presented as a heat map of carbon utilization patterns of 31 different substrates located on the Biolog EcoPlates. Since the most intensive metabolic activity was registered after 120 h, the results obtained at this time are presented in the figures. Average Well Color Development (AWCD) and Shannon–Wiener (H) indices were calculated for all the bottom sediment samples [17].

## 3. Results

### 3.1. Sequencing Data Quality and Diversity Indices

A summary of the sequencing data quality obtained in the current experiment is presented in Table S1 (Supplementary Material). A total of 4,729,922 raw sequences were obtained (1,409,576, 1,401,708, 940,019, and 978,559 for all samples collected in spring, summer, autumn, and winter, respectively). After the filtering step, 3,489,330 sequences remained for further analysis, i.e., approximately 26.2% did not meet the assumed criteria, and poor-quality sequences were removed. After that, denoised F/R quality filtering was performed, which yielded the remaining 3,157,234 (denoised F) and 3,193,392 (denoised R) sequences for all the analyzed seasons. The total number of merged forward-reverse reads was 2,358,157, whereas 2,307,875 sequences remained after removing chimeras. Consequently, the relative number of passed reads oscillated in the range of 36–63% (Table S1).

Based on the NGS results, diversity indices were calculated and summarized in Table S2. Chao1 and ACE are indices estimating the number of species that include both detected and undetected species based on observed species. The analysis of the values of both indices showed an average value of 119.8 in spring, a lower value of 88.7 in summer, and values in the range of 105.7 and 103 in autumn and winter, respectively. Species richness expressed by the H’ index had a mean level of 3.67, 3.41, 3.48, and 3.54 in the bottom sediment samples collected in spring, summer, autumn, and winter, respectively (Table S2). Simpson’s index diversity (1-D) had average values of 0.949 (spring), 0.927 (summer), 0.922 (autumn), and 0.943 (winter). In turn, the comparison of the number of genera detected revealed that the bottom sediments contained on average 124 bacterial genera in spring, 88 in summer, 105 in autumn, and 103 in winter (Table S2).

### 3.2. Seasonal Changes in the Microbiome Structure—Phylum and Class Taxonomic Level

**Figure 3:**
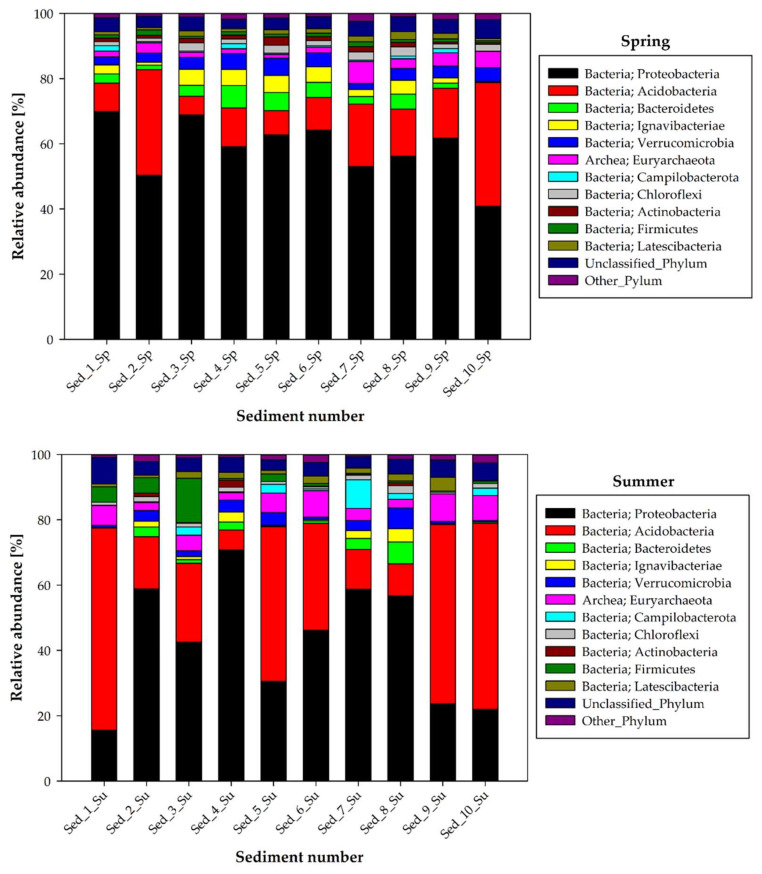

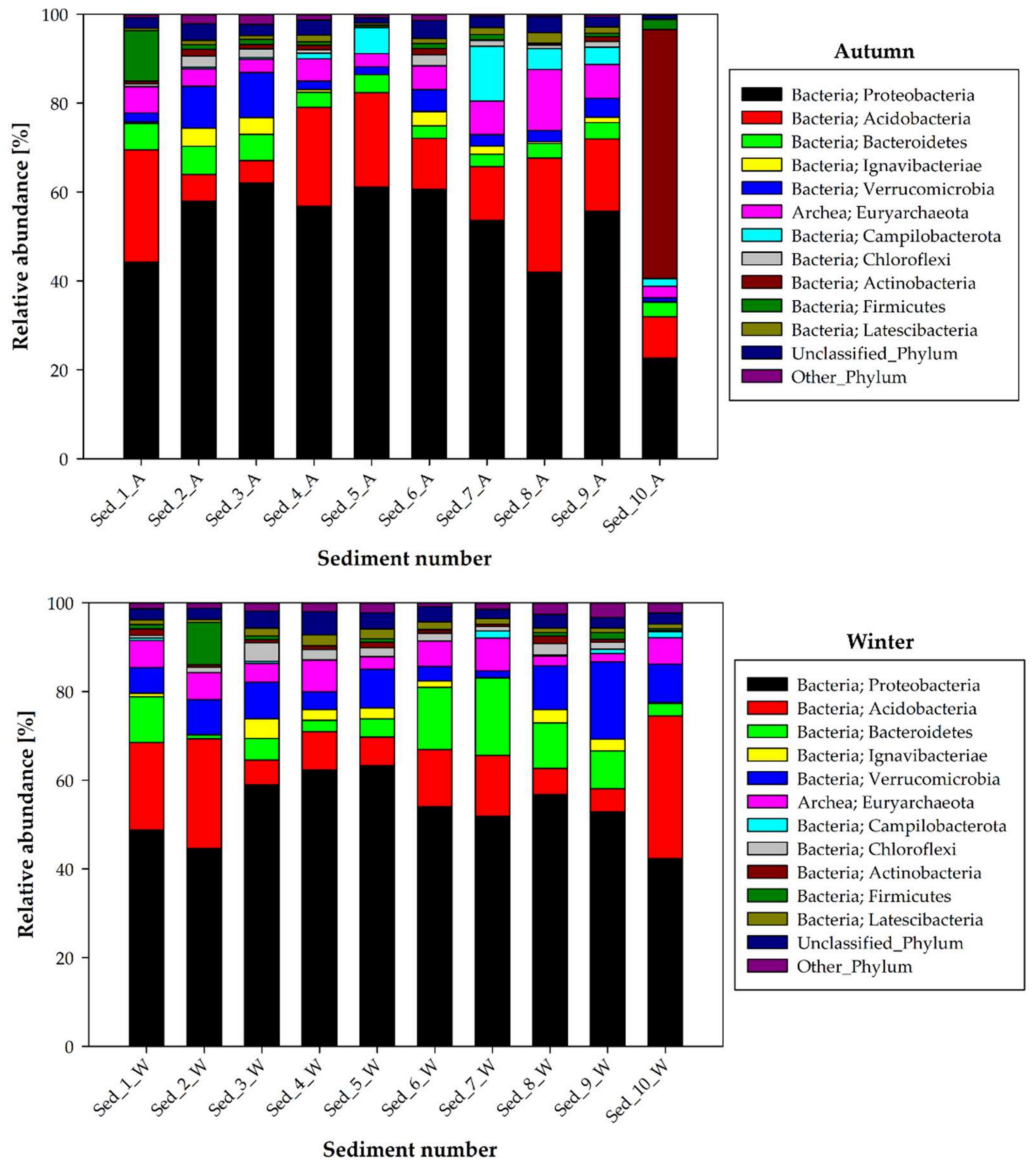
Seasonal changes in the phyla of bacteria in the selected locations of bottom sediment sampling in Cardinal Pond (1–10 Sed—sediment sampling points; Sp—spring, Su—summer, A—autumn, W—Winter).

**Figure 4:**
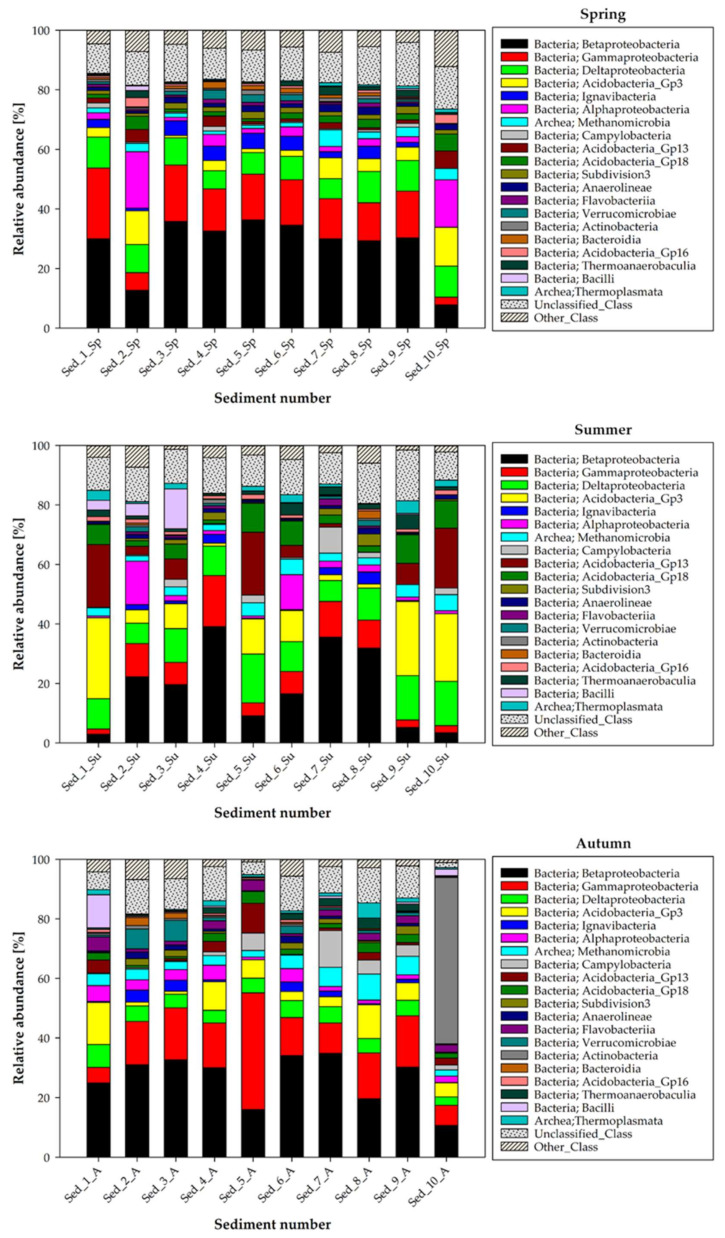

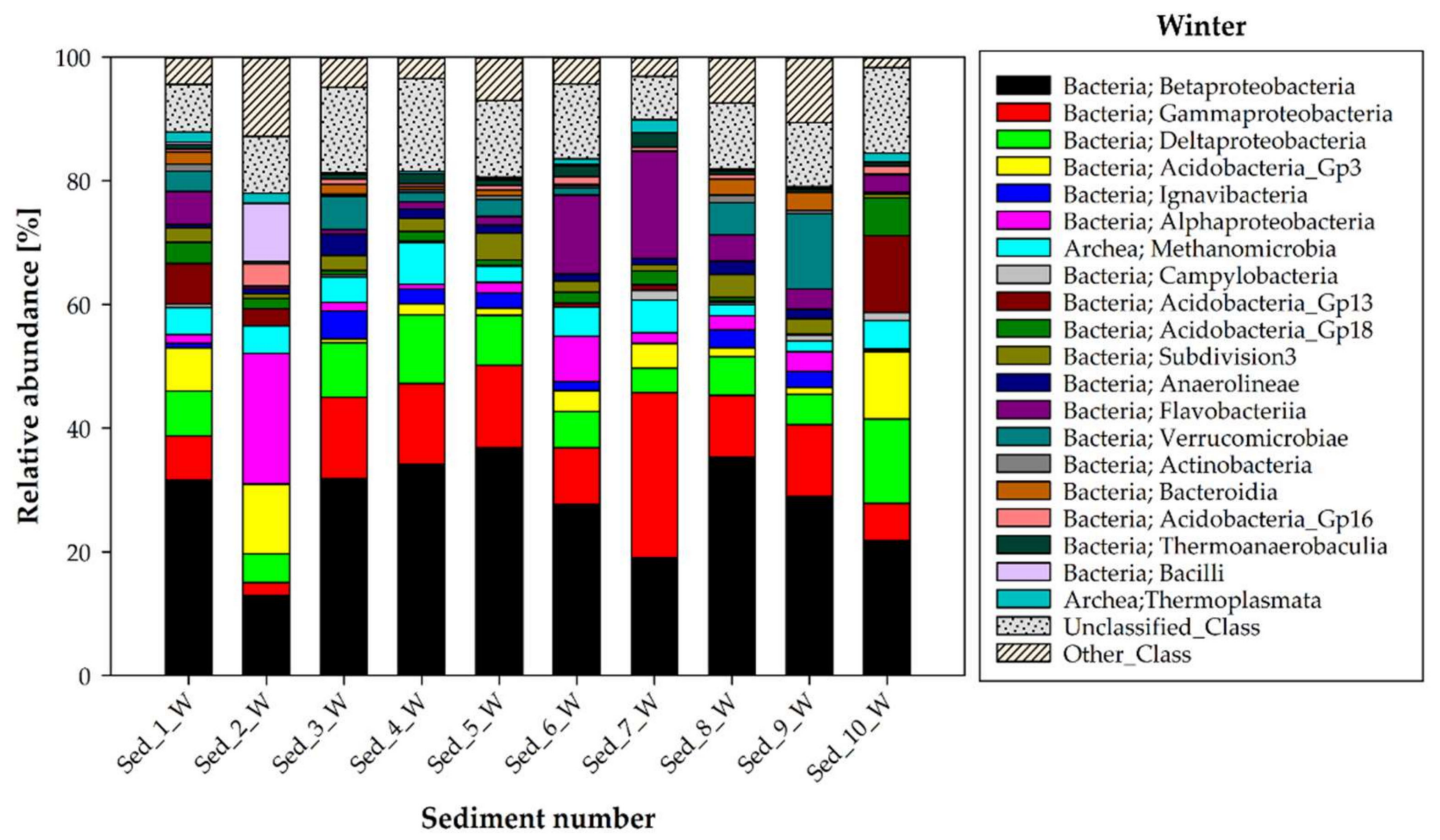
Seasonal changes in the classes of bacteria in the selected locations of bottom sediment sampling in Cardinal Pond (1–10 Sed—sediment sampling points; Sp—spring, Su—summer, A—autumn, W—Winter).

Seasonal changes in the phyla and classes of the bacteria present in the selected locations of the bottom sediments in Cardinal Pond are shown in Figure 3 and Figure 4, respectively, whereas the basic chemical characteristics of the studied bottom sediments, i.e., acidity (pH), redox potential (Eh), and total carbon (TC) in each season, are presented in Table S3. The bottom sediment pH ranged from 6.87 to 7.87, showing spatial and seasonal variation. Much of the sediment had a pH in the range of 7.4–7.7, with a minimum in winter and a maximum in summer or autumn. The Eh value showed a fairly wide range from 126.43 mV to 449.10 mV (Table S3). The seasonal variation of Eh displayed an opposite trend to that of pH. From spring to autumn, the mean Eh values increased linearly (from 264 to 317 mV) and reached a maximum of 325 mV in winter. TC oscillated between 1.11 and 62.05% and depended on sediment location and seasonality, with a maximum in autumn and winter.

The analysis of the bacterial structure at the phylum level indicated that Proteobacteria were dominant in the bottom sediments in spring, accounting for 40.80% to 69.88% of all identified sequences in Sed_10 and Sed_1, respectively (Figure 3).

Acidobacteria were noted as subdominants during the spring season, with relative abundance ranging from 5.72 to 38% at Sed_3 and Sed_10. Bacteroidetes (0.33–6.91%), Verrucomicrobia (2.40–5.28%), and Ignavibacteriae (accounting for up to 5.21%, but their presence was not confirmed at Sed_10) were in the third place in the spring bottom sediment structure (Figure 3). Equally noticeable was the presence of archaeons, represented by Euryarchaeota, which accounted for more than 1% of the sequences at most sampling points and whose relative abundance reached 5.04 and 6.76% in Sed_10 and Sed_7, respectively.

Summer was characterized by the greatest variation in the bacterial structure in the bottom sediment compared to the other seasons (Figure 3). A comparable share of two dominant phyla was noted in most of the bottom sediment samples: Proteobacteria accounted for about 21.9–70.85% of the sequences, while Acidobacteria accounted for 12.2–61.9%. Summer was the time of their most abundant occurrence in the bottom sediment samples. There was also an increase in Firmicutes abundance in summer (0.31–13.52%) compared to spring (0.61–1.70%). Five measurement points also showed a higher Campilobacterota abundance than in spring (0.13–8.76%). A similar trend was confirmed for Euryarchaeota (2.30–8.47%). In turn, the relative abundance of Bacteroidetes was reduced compared to that in spring, accounting for 0.17–2.99% in summer, similarly to Ignavibacteriae (0.31–3.84%).

The relative abundance of Proteobacteria in autumn and winter was in a similar range, accounting for about 22.6–62.05 and 42.34–63.30%, respectively. Similarly, the representatives of Acidobacteria constituted 4.99–25.76% in the autumn period and 5.10–32.15% in winter (Figure 3). Greater differences were noted in the relative abundance of Bacteroidetes, which were more abundant in winter (0.83–17.47%) than in autumn (2.75–6.35%), and Verrucomicrobia (1.48–17.41 and 0.83–10.13%, winter and autumn, respectively). The abundance of Euryarchaeota was higher in autumn (2.63–13.77%) than in winter (1.94–7.47%), as in the case of Campilobacterota (0.12–12.33% autumn and 0.04–1.58% winter)

During autumn and winter, there were also a few exceptions in terms of the abundance of individual bacterial phyla depending on the site of sediment collection for analyses. Thus, in autumn, 11.33% of Firmicutes were recorded in Sed_1, while the abundance of this phylum in the other sediments was in the range of 0.18–2.29% (Figure 3). Analogically, the abundance of Actinobacteria in Sed_10 in autumn amounted to 55.97%, whereas a level of 0.11–1.55% was recorded in the other locations. Additionally, during wintertime, Firmicutes accounted for 9.51% in Sed_2 but extremely lower levels (0.04–1.52%) were recorded in the other sediments.

The differences in the bacterial structure at the class taxonomical level in the bottom sediments are illustrated in Figure 4. In general, the Beta- and Gammaproteobacteria classes predominated in the bottom sediments collected in spring, autumn, and winter. The subdominants at the class level depended on the season in which the sediments were sampled, and thus in spring, the Deltaproteobacteria (6.18–10.50%) and Acidobacteria_Gp3 (0.76–13.05%) classes were noted as subdominants, while Alphaproteobacteria was predominantly present in sediment samples 2 (18.91%) and 10 (15.93%).

The autumn sampling revealed subdominance of the Acidobacteria_Gp3 (1.03–14.13%) and Deltaproteobacteria (2.70–7.71%) classes. Noteworthy, there were two exceptions, namely the class Actinobacteria with the relative abundance of 55.94% in sediment sample 10 and Campylobacteria (12.33%) in Sed_7, which were dominant in the structure of the bottom sediment microbiome (Figure 4).

The subdominance of the Acidobacteria_Gp3 (0.65–11.20%) and Deltaproteobacteria (4.02–13.61%) classes was also confirmed in winter; however, it should be noted that the significant contribution of the Alphaproteobacteria class was confirmed especially in Sed_2 and Sed_6, where the relative abundance of representatives of this class was 21.15% and 7.65%, respectively. Additionally, incidentally but with high relative abundance in the selected sediments samples, representatives of Flavobacteriia (12.70–17.27% in Sed_6 and Sed_7) and Verrucomicrobiae (5.37–12.17% in Sed_3, Sed_8, and Sed_9) were recorded.

The analysis of the microbiome structure in the bottom sediments from Cardinal Pond at the class level revealed that the summer season significantly modified the bacterial composition, which was dramatically different from that in the other seasons (Figure 4). The dominance of the Betaproteobacteria class (16.49–39.14%) was shown only in some sediments numbered 2–8. In the other sediment sampling points, there was an increase in the proportion of Acidobacteria_Gp3 (10.99–27.19%) and Deltaproteobacteria (7.02–14.90%), while the proportion of Gammaproteobacteria in comparison with the other seasons was lower (1.77–11.17%). The relative abundance of the Acidobacteria_Gp13 (0.16–21.20%) and Acidobacteria_Gp18 (1.21–9.78%) classes also increased during the summer in relation to the other seasons of the year.

The statistical analysis of samples taken in summer and spring revealed statistically significant differences in the relative abundance of 18 classes, with 14 classified (Figure S1). In the analysis of summer vs. autumn and summer vs. winter samples, it was 17 classes. The same analysis was carried out at the genus level (Figure S2). The highest number of statistically significant differences was observed between samples taken in summer and winter (117; 42.545% of all analyzed genera). In the comparison of summer samples and spring samples, it was 108 genera (38.163% of all analyzed genera) and summer vs. autumn—106 genera (38.129% of all analyzed genera).

Regardless of the season, the bottom sediments showed the presence of the class Methanomicrobia, which reached the level of 1.08–5.51% in spring, 1.95–5.45% in summer, 2.09–8.73% in autumn, and 1.69–6.81% in winter (Figure 4).

### 3.3. Seasonal Changes in the Microbiome Structure—Genus Taxonomic Level

**Figure 5:**
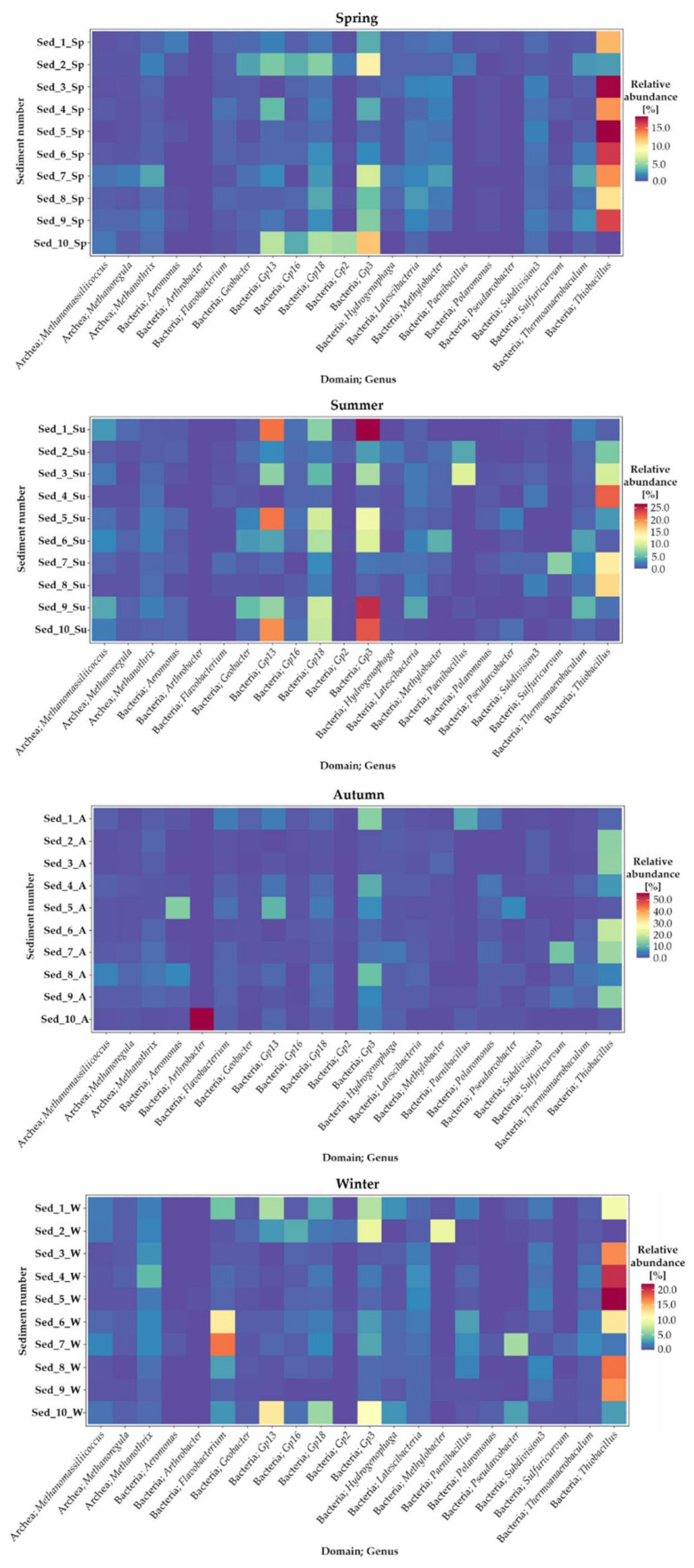
Heat map illustrating seasonal changes in the genera of bacteria in the selected locations of bottom sediments in Cardinal Pond (1–10 Sed—sediment sampling points; Sp—spring, Su—summer, A—autumn, W—Winter).

Seasonal changes in the identified genera of bacteria present in the selected locations of bottom sediment sampling in Cardinal Pond are presented as heat maps in Figure 5.

It was evident that both the season and the sampling location modified the bacterial structure in the analyzed bottom sediment samples. The greatest relative abundance of bacteria that were classified to the taxonomic level of the genus was recorded in spring, summer, and winter.

Generally, *Thiobacillus* dominated in the bottom sediments in the spring period. They constituted c.a. 18% (Sed_3 and Sed_5), 16.60% (Sed_6 and Sed_9), 13.60% (Sed_4 and Sed_7), and 10.71–12.42% (Sed_8 and Sed_1, respectively). In summer, the relative abundance of *Thiobacillus* was 16.36–22.22% (Sed_8 and Sed_4), 6.06–9.85% (Sed_2 and Sed_3), and 0.28–3.45% in the other sampling points (Figure 5). In turn, the *Thiobacillus* abundance in winter amounted to c.a. 21% (Sed_4 and Sed_5), 16.50% (Sed_3, Sed_8 and Sed_9), and 9.84–12.37% (Sed_1 and Sed_7). During autumn, *Thiobacillus* constituted c.a. 19% (Sed_6) and 14% (Sed_2, 3, 7 and 9), whilst the highest abundance in this period was exhibited by *Arthrobacter*, which reached the value of 55.73% in sed_10.

In the structure of bacteria identified at the genus level, the representatives of Gp3 bacteria were indicated as subdominants in all seasons, accounting for approx. 0.82–26.76% in summer, 1.04–13.80% in autumn, and similar levels of 0.54–11.88% and 0.41–10.96% in spring and winter, respectively (Figure 5). Additionally noteworthy were the representatives of Gp 13 and Gp 18, which were abundant in the microbiome of bottom sediments as well. In spring, they accounted for 0.62–5.92 and 0.94–5.63%, respectively. In summer, their relative abundance increased to 1.13–21.20% (Gp3) and 1.21–9.78% (Gp 18). They represented 0.68–4.44 and 0.69–6.99%, respectively, in autumn and 0.20–12.39 and 0.60–6.11% in winter. The incidental presence of the genera *Aeromonas* (13.49% in Sed_5 and 5.05% in Sed_8) and *Pseudomonas* (12.36% in Sed_5) in autumn is noteworthy as well.

**Figure A1:**
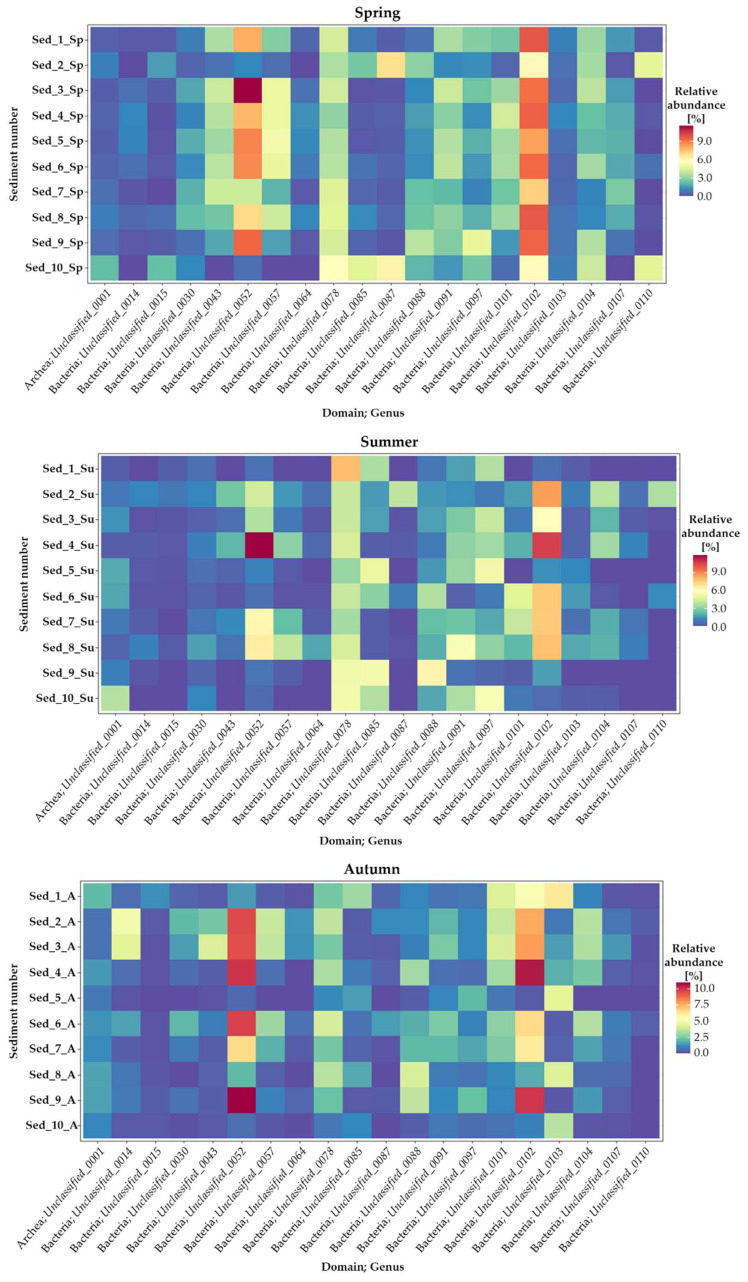

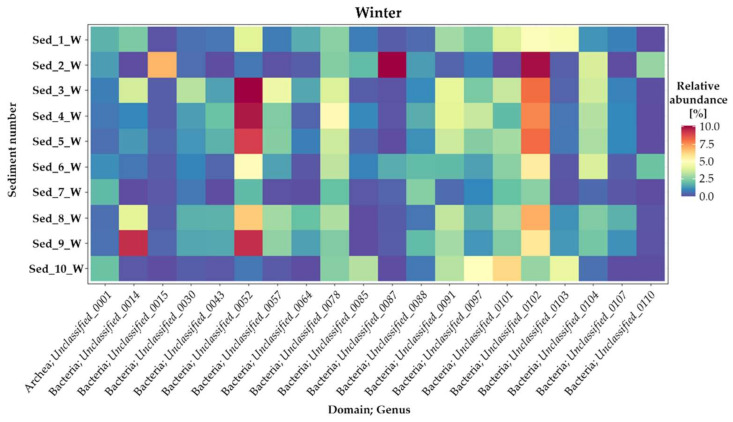
Heat maps illustrating seasonal changes in the unclassified genera of bacteria in the selected location of the bottom sediments in Cardinal Pond (1–10 Sed—sediment sampling points; Sp—spring, Su—summer, A—autumn, W—Winter).

The description of the bottom sediment microbiome should also emphasize the presence of genera described as unclassified (Figure A1; Appendix A). In this group, the highest frequency in most of the sampling points in Cardinal Pond was exhibited by unclassified_0052 and unclassified_0102 regardless of the sampling season. The relative seasonal abundance of unclassified_052 fluctuated around 11% on average, while the unclassified_0102 group accounted for 9–10% (Figure A1). The Archaea community in the bottom sediments was represented by c.a. 2.5–3.0% of unclassified_0001.

### 3.4. Seasonal Changes in the Functional Activity of Bacteria Inhabiting Bottom Sediments

**Figure 6:**
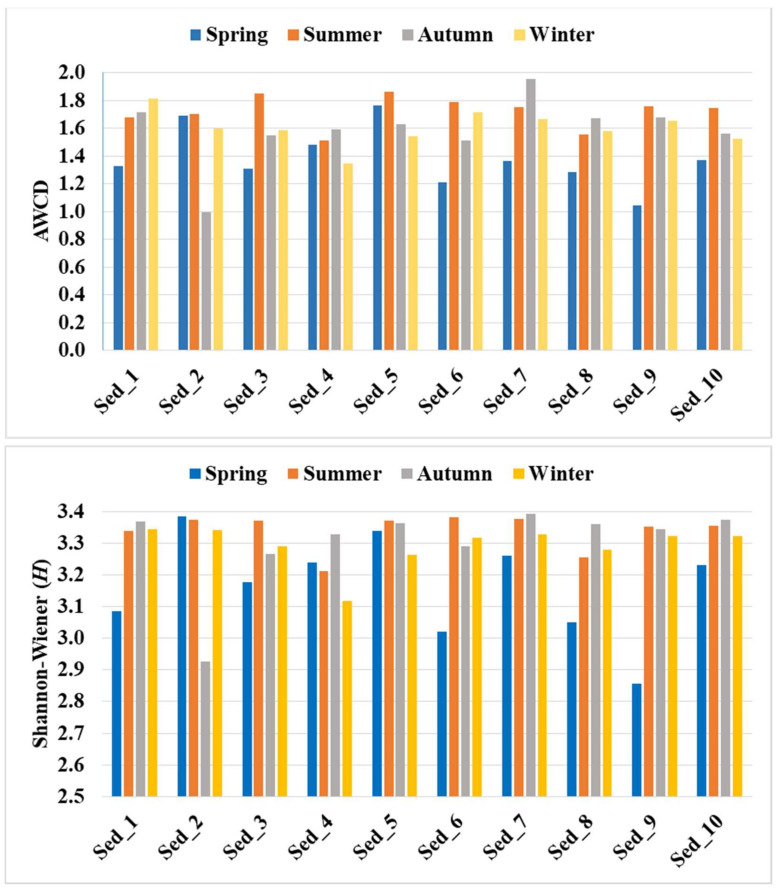
Average Well Color Development (AWCD) and Shannon–Wiener (H) levels in 10 bottom sediment sampling points in the four seasons.

As demonstrated in Figure 6, the highest microbial activity expressed by the AWCD values (as high as 1.72) was recorded in summer, followed by winter (c.a. 1.60) and autumn (averagely 1.58), whereas the lowest metabolic activity was noted in the samples collected in spring (c.a. 1.38).

The calculated Shannon–Wiener index of functional diversity reached the highest mean level (H = 3.34) in the sediments collected in summer. Slightly lower levels were recorded in autumn and winter (H = 3.30), and the lowest level was noted in spring (H = 3.16).

**Figure 7:**
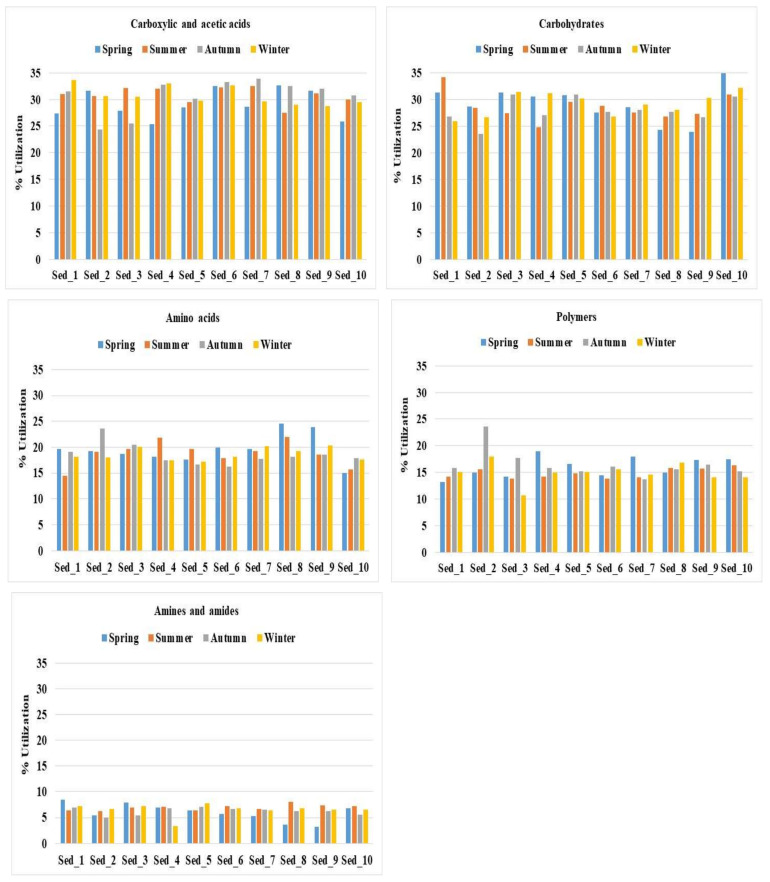
Total carbon source utilization response (%) in 10 bottom sediments sampled during spring, summer, autumn, and winter for the different carbon substrate groups.

The catabolic diversity of the microbial community evaluated by substrate utilization in the Biolog EcoPlate incubated for 120 h is demonstrated in Figure 7. Similar to the biodiversity structure, the metabolic activity was influenced by the season of the year.

It was found that carboxylic and acetic acids were the most preferable source of carbon for the bottom sediment microorganisms. The compounds were utilized at an average level of over 30% in the samples collected during summer, autumn, and winter. In turn, the utilization of these carbon sources in spring amounted to c.a. 29.21% (Figure 7). The next most readily utilized compounds were carbohydrates, with an average consumption of about 29% in the sediments collected in spring and winter and 27–28% in autumn and summer. Amino acids were the third most preferably metabolized carbon source in spring (19.63%), while their metabolism in the other seasons was just above 18%. The consumption of polymers by the bottom sediment microbiome was estimated to be 16.52% in autumn, 16% in spring, and approx. 14.9% in summer and winter. Amines and amides were found to be the least readily used carbon sources, with 6.9% consumption in summer, 6.5% in autumn and winter, and 5.9% in spring (Figure 7).

**Figure A2:**
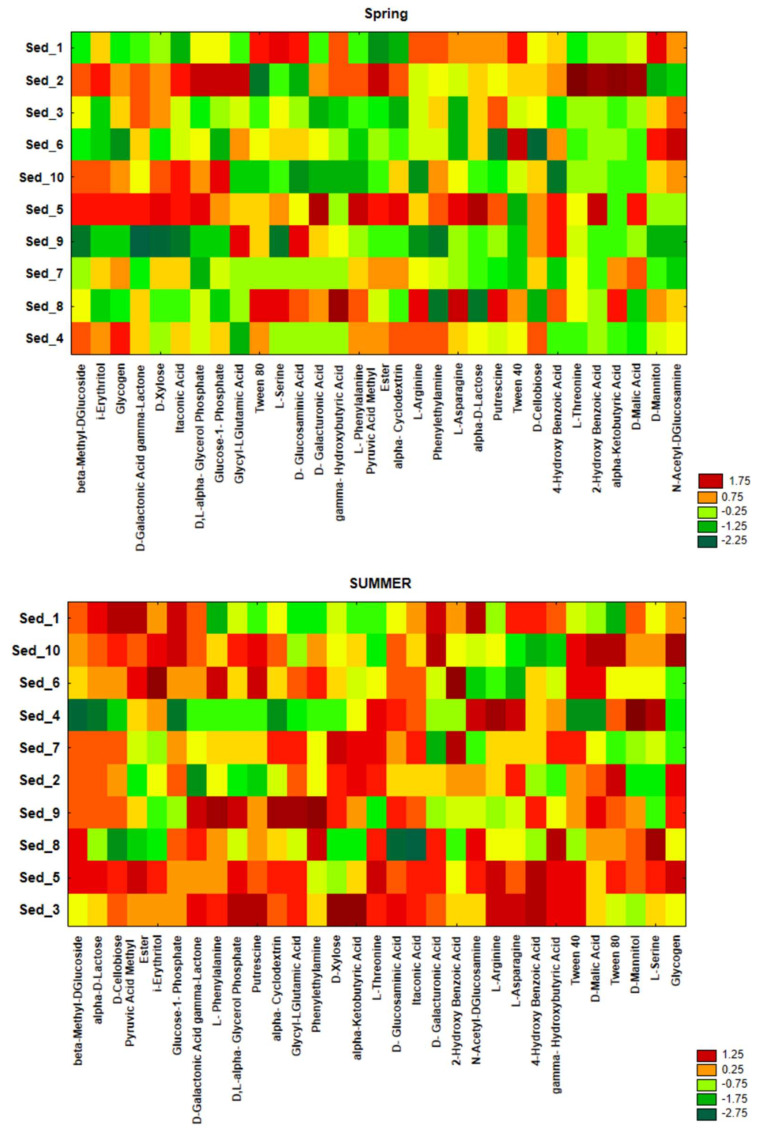

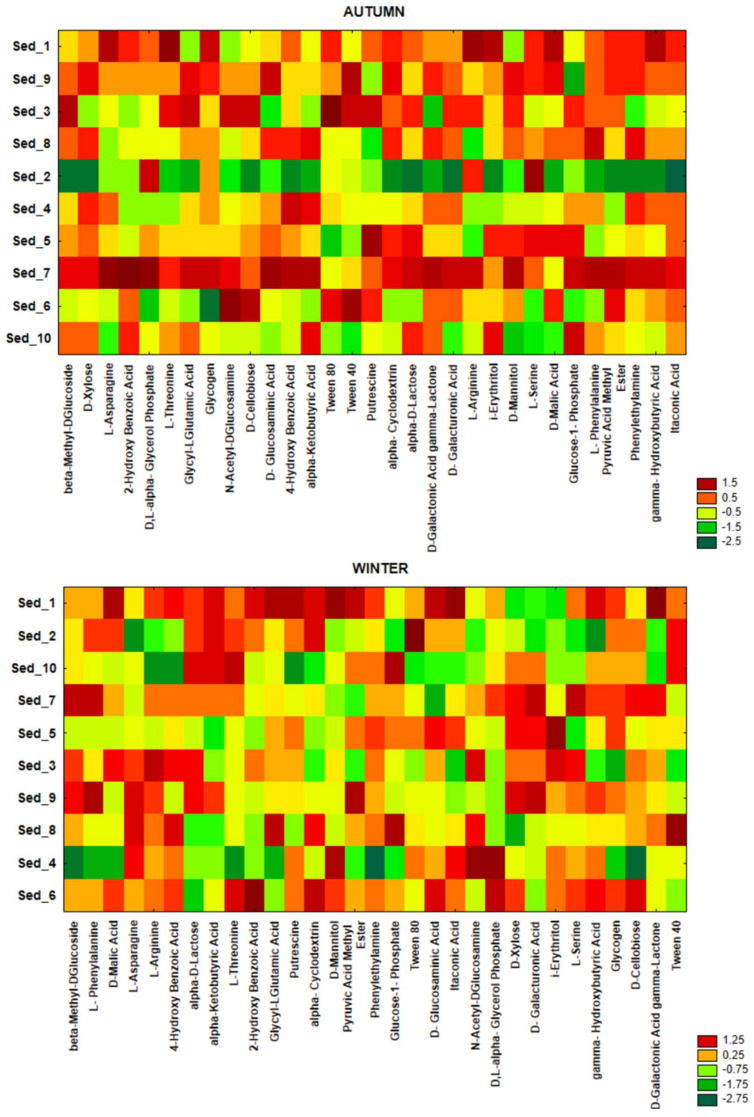
Heat maps of carbon utilization patterns of 31 substrates from the Biolog EcoPlates incubated for 120 h with the bottom sediments in Cardinal Pond. The results are visualized as standardized data of absorbance measurement at a wavelength of 590 nm (higher values mean higher functional activity).

The data provided by the CLPP technique were also visualized as heat maps of carbon utilization patterns of 31 substrates from the Biolog EcoPlates (Figure A2). The results were visualized as standardized data of absorbance measurement at a wavelength of 590 nm with an assumption that higher values mean higher functional activity. The most intensive processes of carbon utilization in the bottom sediment environment were found in summer, autumn, and winter in contrast to spring, when the processes were less intensive. For example, beta-Methyl-DGlucose, D-Cellobiose, Glucose-1-Phosphate, alpha-Cyclodextrin, and L-Threonine were the most intensively metabolized sources of carbon in the bottom sediments sampled in summer (Figure A2).

### 3.5. Bacterial Functional Annotation

**Figure 8:**
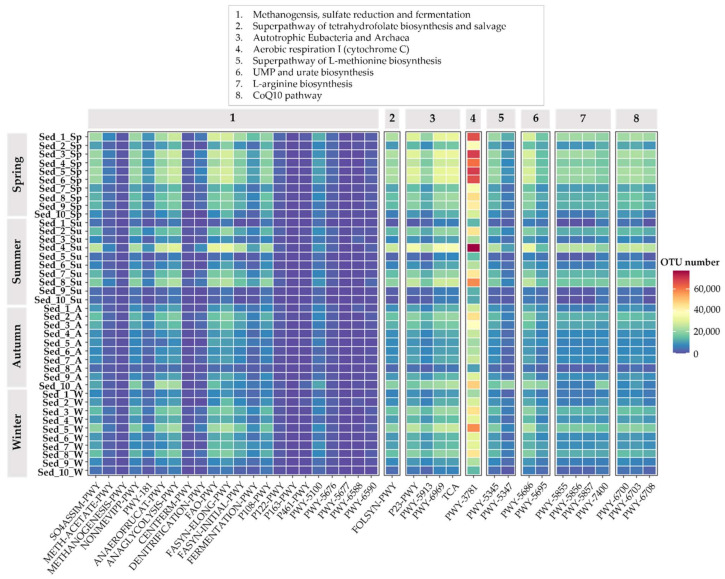
Heat map of the pathways annotated by eight pathway clusters (1–8).

The Kyoto Encyclopedia of Genes and Genomes (KEGG) knowledge base was used to predict the ecological function of bacterial community genes in the different points in the pond according to the results of classification of the 16S rRNA sequences. It was found that, in general, the relative abundance of ecological function genes related to carbon (*p* = 0.000411) and sulfur (*p* = 0.0000) was significantly higher than those related to nitrogen and phosphate, with the exception of spring when the highest relative abundance of ecological function genes related to nitrogen was noted in comparison to all the other seasons (*p* = 0.0399; Figure 8). This indicates that both the carbon and sulfur cycles in the surface sediments of the king carp farming pond may be stronger than the nitrogen and phosphorus cycles. What is more, a higher number of metabolic pathways were detected by the KEGG database in Sed_1–6 collected in spring than in the other seasons, which indicates that the functional activities of the microorganisms are very high during this period of the year. As evidenced by our results, aerobic respiration I with the use of cytochrome C is one of the main metabolic pathways used by microorganisms colonizing the bottom sediments (Figure 8).

Lesser alterations were observed in fermentation metabolism than in the autotrophic Eubacteria and Archaea metabolism. The predicted bacterial fermentation metabolism, for example, denitrification, homolactic fermentation, glycolysis III, and mixed acid fermentation, was detected in the members of the active and total community compositions in all seasons, but particular intensification of these processes was noted in spring (Figure 8). Similarly, TCA cycle VI (obligate autotrophs) and reductive TCA cycles I, V, and VI were more frequently observed in the total community compositions in spring than in the other seasons. We also analyzed the CoQ10 pathway characteristic for prokaryotes and evidenced its maximum in spring in comparison with the other seasons. In the present study, we predicted L-arginine biosynthesis IV (archaebacteria) pathways in the total bacterial community. In detail, the relative abundance of L-arginine biosynthesis IV (archaebacteria) pathways showed the highest abundance in the open water habitats and the lowest abundance in the pond shoreline (Figure 8).

## 4. Discussion

The major goal of this study was to identify (using culture-independent techniques) the composition of the bacterial microbiome, to determine seasonal changes in the structure of autochthonous bacteria inhabiting the bottom sediments from Cardinal Pond in Ślesin, and to estimate their metabolic and functional activities.

Using the NGS technique with the Illumina MiSeq platform, bacterial diversity was determined at different taxonomic levels, from phyla to genera [24]. Importantly, the state-of-the-art sequencing technology used allowed us to identify the bacterial microbiome within a group of viable but non-culturable bacteria, which greatly enhances the cognitive value of this paper and sheds new light on the bacteria present in the bottom sediments, which still remain rather relatively unrecognized.

Environmental factors are known to shape the structure and function of microbial communities [9]; hence, the location of the bottom sediment sites in the study pond and the time of the year (indirectly temperature) influenced the presence of autochthonous bacteria, as demonstrated in the current study.

The analysis of the bacterial structure at the phylum level revealed the dominance of Proteobacteria inhabiting the bottom sediments in a large abundance (15–70%) in all seasons of the year, with prevalence in spring (Figure 3) at all sampling points in Cardinal Pond (Figure 2). Here, it is worth noting that Proteobacteria are heterotrophic, versatile opportunists and have been extensively studied not only as both pathogens and beneficial symbionts of plants and animals, but also as ubiquitous organisms that live freely (autochthonously) in many environments [25]. In addition, they find potential applications in biotechnology as iron-oxidizing bacteria [26] and have an underestimated potential to produce bioactive molecules [27]; therefore, their presence in bottom sediment seems to be desirable. Acidobacteria were the second dominant bacterial phylum after Proteobacteria. They were present at every bottom sediment collection point in all seasons (Figure 3), with an outstanding relative abundance characterized by seasonal variation, especially in summer (9.80–62%). It has been indicated that this phylum includes bacteria that are ubiquitous and abundantly distributed in most ecosystems [28], thus their abundant presence in the bottom sediments is not surprising. Acidobacteria is an ecologically important phylum with a set of genes involved in various metabolic pathways. It plays a dynamic role in ecological processes, namely regulation of biogeochemical cycles, degradation of biopolymers, and secretion of exopolysaccharides [28]. The third most abundant bacterial phylum identified in the bottom sediment in the current study was Bacteroidetes, with predominance in winter (c.a. 2.50–17.50%). The Bacteroidetes phylum appears to be dominant in the soil environment, where it indicates soil “fatigue” [29], and in human and animal intestines [30]. These bacteria thrive on their ability to secrete a variety of carbohydrate-active enzymes (CAZymes) that act on the highly diverse glycans in the soil [29]. Importantly, soil Bacteroidetes are less well studied than the human gut symbionts; however, increasing numbers of studies are exploring the important biochemical and physiological phenomena associated with these bacteria [30].

Betaproteobacteria was the most abundant class of bacteria identified in the current study (Figure 4). Indeed, its representatives occurred at similar abundance levels in all seasons and in all bottom sediment locations, indicating that they are ubiquitous bacteria [31] and prefer bottom sediments of water bodies as niches for colonization. In addition, they are abundantly found in drinking water, including mineral water [32]. They are potential but sometimes overlooked opportunistic pathogens that can be transmitted through water and aqueous solutions [33]. In addition, some Betaproteobacteria present in drinking water with inherent and sometimes acquired antibiotic resistance, carrying virulence factors, and present in biofilm structures can persist even after water disinfection and reach the consumer [32]. The class Gammaproteobacteria was also recorded in all seasons and all bottom sediment sampling points (Figure 4). Their presence is ecologically important, as they play an important role in nutrient cycling in coastal marine ecosystems [33], in addition to being responsible for carbon fixation in coastal sediments [34] and having metal-reducing abilities [35]. The class Deltaproteobacteria was most abundantly recorded in the bottom sediments from Cardinal Pond during summer (Figure 4); nonetheless, their presence was confirmed in all seasons. Bacteria of this class are important members of the marine microbiota with diverse capabilities of reductive dehalogenation, respiration of organohalogens (halogenated compounds that contain at least one halogen (fluorine (F), chlorine (Cl), bromine (Br), or iodine (I)) bound to carbon), thus playing an important role in the natural cycling of organohalogens in the environment [36].

The highest bacterial diversity in the bottom sediments from Cardinal Pond was observed at the taxonomic level of genera (Table 1), with the indication that the presence of individual bacterial genera varied with the seasons (Figure 5). Considering the presence of all genera identified in the current study, the highest biodiversity of bacteria in the bottom sediments was shown in autumn and winter (Table A1). Some genera noted in autumn were not present in other seasons of the year, i.e., *Aeromonas*, *Arthrobacter*, *Acinetobacter*, *Polaromonas*, *Methylobacter*, *Flavobacterium*, etc., whereas the presence of, e.g., *Shewanella* and *Iodobacter* was detected only in winter (Table A1). In terms of the sediment collection points, most genera in all seasons were identified in sediments Sed_1, Sed_5, Sed_7, Sed_9, and Sed_10.

In general, *Thiobacillus* was the undisputed generic dominant in the bottom sediments, regardless of the season. Its contribution to the bacterial structure was significant at most of the analyzed measurement points in Cardinal Pond. *Gp3*, *Gp13*, *Gp18* dominated in summer, likewise in autumn, together with representatives of *Polaromonas, Flavobacterium*, and *Thermoanaerobaculum*. In turn, *Flavobacterium*, *Gp3*, and *Gp13* occurred most frequently in winter and in the greatest number of locations (Table A1). All these bacteria are ecologically important genera, i.e., *Thiobacillus*, which is widespread in marine and terrestrial habitats, oxidizes sulfur, producing sulfates and generates sulfuric acid in deep ground deposits that dissolve metals in mines [37]. *Polaromonas* are among the dominant bacteria of glacial ice and sediments worldwide [38] with the ability to oxidize a wide array of unusual energy sources, including H_2_ [39], arsenite [40], and a broad range of recalcitrant organic compounds [38]. With these characteristics, *Polaromonas* are referred to as metabolically diverse “opportunitrophs” [41] that take advantage of transient periods of higher temperatures and substrate availability occurring in extreme environments. *Flavobacterium* and *Thermoanaerobaculum* are able to hydrolyze a wide variety of organic compounds, e.g., several carbohydrates and biomacromolecules [42], thus their presence in bottom sediments is desirable. Acidobacteria subgroups *Gp3*, *Gp13*, and *Gp18* are usually the best known for the most positive correlations with gene families associated with carbon degradation, especially those involved in hemicellulose degradation [43].

The analysis carried out with the use of the Biolog EcoPlate system evidenced similar trends as NGS (with respect to biodiversity) in the variability of metabolic activities under the influence of the season, which indicates different dynamics of microbiological activity in the sediments during the year. By combining both analytical techniques, it was possible to identify the highest and lowest catabolic activity and changes in the structure of the bacterial microbiome in the bottom sediments from the culture pond. AWCD and H reached the highest level in summer, followed by winter, autumn, and spring, which clearly indicates the highest peak of metabolic activity and biodiversity in summer and winter, while the beginning of the growing season (spring) is the time of the lowest microbial activity in the bottom sediments. The analysis of the preferences of the microorganisms present in the bottom sediments for utilization of carbon sources revealed that carboxylic and acetic acids and carbohydrates were metabolized most readily (in about 29–30%), followed by amino acids (about 20%), while amines and amides were metabolized the least readily (in about 7%).

Only a few studies on the metabolic activity of bottom sediments performed using the Biolog EcoPlate system are currently available, hence the results presented in this paper expand the knowledge in this area, as they were obtained through year-long monitoring of the bacterial activity. Zhao, et al. [44] studied the functional diversity of the bacterial microbiome in water and sediment from shrimp ponds and found that the average value of absorption of the carbon sources utilized by the microorganisms in the sediment was significantly higher than that found in the water samples. The researchers [44] reported an H value in the sediment samples in the range of 3.015–3.28, which is in agreement with our observations and the H value of 3.16–3.34. However, preferences for carbon utilization by microorganisms reported by Zhao, et al. were different from our findings, as they noted that amino acids and polymers were the most preferable carbon sources [44]. In turn, similar patterns in carbon utilization to those noted in the current study were evidenced in a horizontal subsurface-flow constructed wetland, where higher utilization of carbohydrates, carboxylic acids, and amino acids was noted in upper front substrate microorganisms than in lower back substrate microorganisms [45]. Additionally, Wu, et al. [46] observed that microorganisms originating from bottom sediments of the freshwater lake Taihu in China metabolized carboxylic and acetic acids and carbohydrates most frequently after polymers. It was also noted that the sediment microbial communities of lakes in summer and autumn showed more versatile substrate utilization patterns than in spring [47], which was revealed in the current study. In general, the lake and river sediment microbiome in summer and autumn preferred to use carbohydrates [46], likewise in our study.

The bacterial functional analysis performed with the use of the KEGG tool confirmed that aerobic respiration I with the use of cytochrome C was the main pathway used by the microbiome of the studied bottom sediments. In this context, Hamada, et al. [48] revealed that cbb3 oxidases (cytochrome C), which are commonly known as aerobic respiratory enzymes, were involved in denitrification and influenced the lifestyle of *Psedomonas aeruginosa* PAO1 in anoxic conditions. We evidenced that the investigated bottom sediments from Cardinal Pond were characterized by the highest relative abundance of bacteria capable of using CoQ10 pathways. George, et al. [49] indicated that most Gram-negative facultative anaerobes use CoQ10 pathways, and these pathways can be identified in some anoxygenic phototrophic bacteria (isolated from lake sediment). Additionally, the presence of Gram-negative facultative anaerobes may correlate with temporary flooding and drying of the pond [49].

## 5. Conclusions

The combination of two research techniques (NGS and CLPP) resulted in the identification of changes in the structure of bacteria inhabiting bottom sediments and their functional activity under the influence of seasons. Thus, the study provided new knowledge of the still rather poorly explored microbiome of bottom sediments. Both the location of sampling in Cardinal Pond and the season of the year had an impact on its biodiversity and metabolic activity. Proteobacteria, Acidobacteria, and Bacteroidetes were identified as dominant phyla, followed by Euryarchaeota, Firmicutes, Ignavibacteriae, Campilobacterota, Chloroflexi, and Actinobacteria. Beta- and Gammaproteobacteria were the most abundantly represented classes, but Delta- and Alphaproteobacteria, Acidobacteria-Gp3, Acidobacteria-Gp13, and Acidobacteria-Gp18 were also present with fairly high frequency, depending on the season. At the taxonomic level of genera, the following representatives dominated: *Thiobacillus* and *Gp3* in spring, *Thiobacillus*, *Paenibacillus*, *Gp3*, and *Gp13* in summer, *Thiobacillus*, *Gp3*, *Aeromonas*, *Arthrobacter*, and *Pseudomonas* in autumn and *Thiobacillus*, *Gp3*, *Gp13*, *Flavobacterium,* and *Shewanella* in winter. Compared to the other seasons, the greatest modifications of the bacterial microbiome structure were observed in summer. Similarly, the highest metabolic activity of bacteria inhabiting bottom sediments was found in summer. It was expressed by the AWCD index, which reached the following gradient: summer > winter > autumn > spring (analogically to the H index). The microbial preferences for the utilization of carbon sources were as follows: carboxylic and acetic acids > carbohydrates > amino acids > polymers > amines and amides. Aerobic respiration I with the use of cytochrome C was the main pathway used by the microbiome of the studied bottom sediments.

## Figures and Tables

**Table 1:** Seasonal changes in the number of dominant bacteria in the bottom sediments based on taxonomic affiliation: phylum-class-genus.

Taxonomic Affiliation	Seasonal Variation in Abundance				Main Dominants
	Spring	Summer	Autumn	Winter	
Phyla	13	14	12	15	Proteobacteria Acidobacteria Bacteroidetes Euryarchaeota
Classes	28	28	22	29	Betaproteobacteria Gammaproteobacteria Deltaproteobacteria Methanobacteria
Genera	28	28	35	39	Table A1 (Appendix A)

## Data Availability

The data presented in this study are available on request from the corresponding author.

## Supplementary Materials

The following supporting information can be downloaded at: https://www.mdpi.com/article/10.3390/biology11060913/s1, Figure S1: Metastat analysis showing significant differences in bacteria composition (class level) in smples from summer compared to other analyzed sea, Figure S2: Metastat analysis showing significant differences in bacteria composition (genus level) in samples from summer compared to other analyzed sea, Table S1: Sequencing data quality, Table S2: Diversity indices (NGS), Table S3: Basic characteristic of bottom sediments.

## Appendix A

**Table A1:** Genus-classified bacteria present in the bottom sediments and their seasonal variation (+ indicates the presence of a particular genus).

Sample	1	2	3	4	5	6	7	8	9	10
Genus	SPRING									
*Thiobacillus*	+	+	+	+	+	+	+	+	+	
*Serratia*									+	
*Methylobacter*							+			
*Thermoanaerobaculum*		+					+		+	
*Geobacter*		+								
*Gp2*										+
*Gp3*	+	+		+			+	+	+	+
*Gp13*		+		+						+
*Gp16*		+								+
*Gp18*		+					+	+	+	+
	SUMMER									
*Thiobacillus*		+	+	+	+		+	+		
*Paenibacillus*		+	+							
*Geobacter*					+	+			+	
*Trichococcus*	+									
*Thermoanaerobaculum*	+					+	+		+	
*Pseudoarcobacter*					+					
*Phenylobacterium*						+				
*Methylobacter*						+				
*Malikia*							+			
*Gp3*	+	+	+		+	+			+	+
*Gp13*	+	+	+		+	+			+	+
*Gp18*	+		+		+	+	+	+	+	+
	AUTUMN									
*Aeromonas*					+			+		
*Arthrobacter*										+
*Acinetobacter*					+					
*Pseudomonas*					+			+		
*Thiobacillus*	+	+	+	+		+	+	+	+	
*Sulfuricurvum*							+		+	
*Paenibacillus*	+									
*Polaromonas*	+			+	+		+			
*Pseudoarcobacter*					+					
*Thermoanaerobaculum*				+		+	+	+	+	
*Rheinheimera*					+			+		+
*Methylobacter*			+							
*Masillia*					+					
*Luetolibacter*			+							
*Hydrogenophaga*							+			
*Flavobacterium*	+				+		+	+	+	
*Deefgea*								+		
*Gp3*	+			+	+	+	+	+	+	+
*Gp13*	+			+	+			+		+
*Gp18*	+			+	+			+	+	
	WINTER									
*Flavobacterium*	+					+	+	+		+
*Shewanella*							+			
*Thiobacillus*	+		+	+	+	+		+	+	+
*Pseudomonas*							+			+
*Paenibacillus*		+								
*Thermoanaerobaculum*							+			
*Polaromonas*						+	+			
*Luteolibacter*									+	
*Iodobacter*							+			
*Hydrogenophaga*	+									+
*Gp3*	+	+				+	+			+
*Gp13*	+	+								+
*Gp16*		+								
*Gp18*	+						+			+
