# Supplementary figures and images for: Functional and Seasonal Changes in the Structure of Microbiome Inhabiting Bottom Sediments of a Pond Intended for Ecological King Carp Farming
Source: Biology (Basel). 2022 Jun 14;11(6):913. doi: 10.3390/biology11060913 (PMC9220171; doi:10.3390/biology11060913)

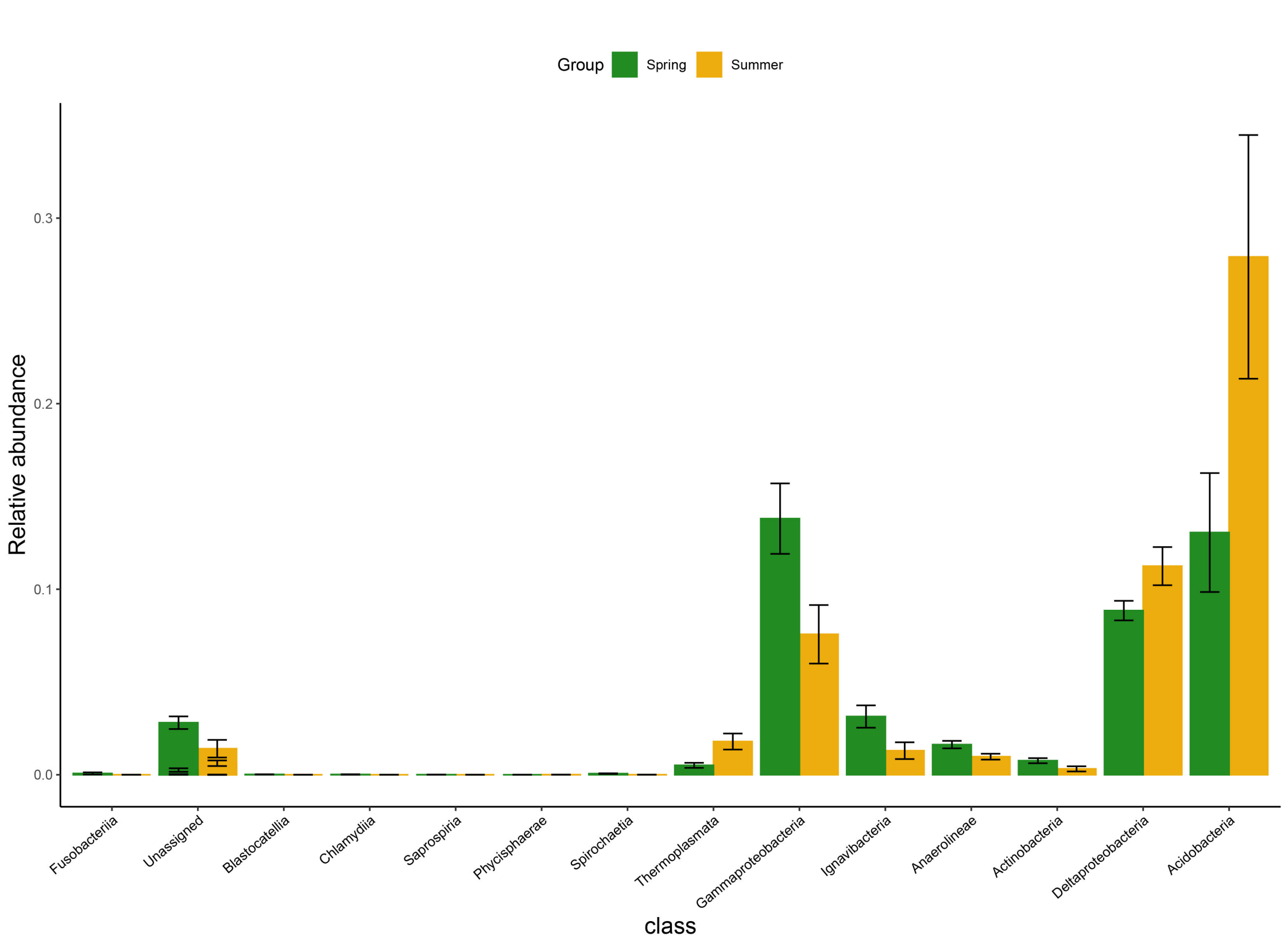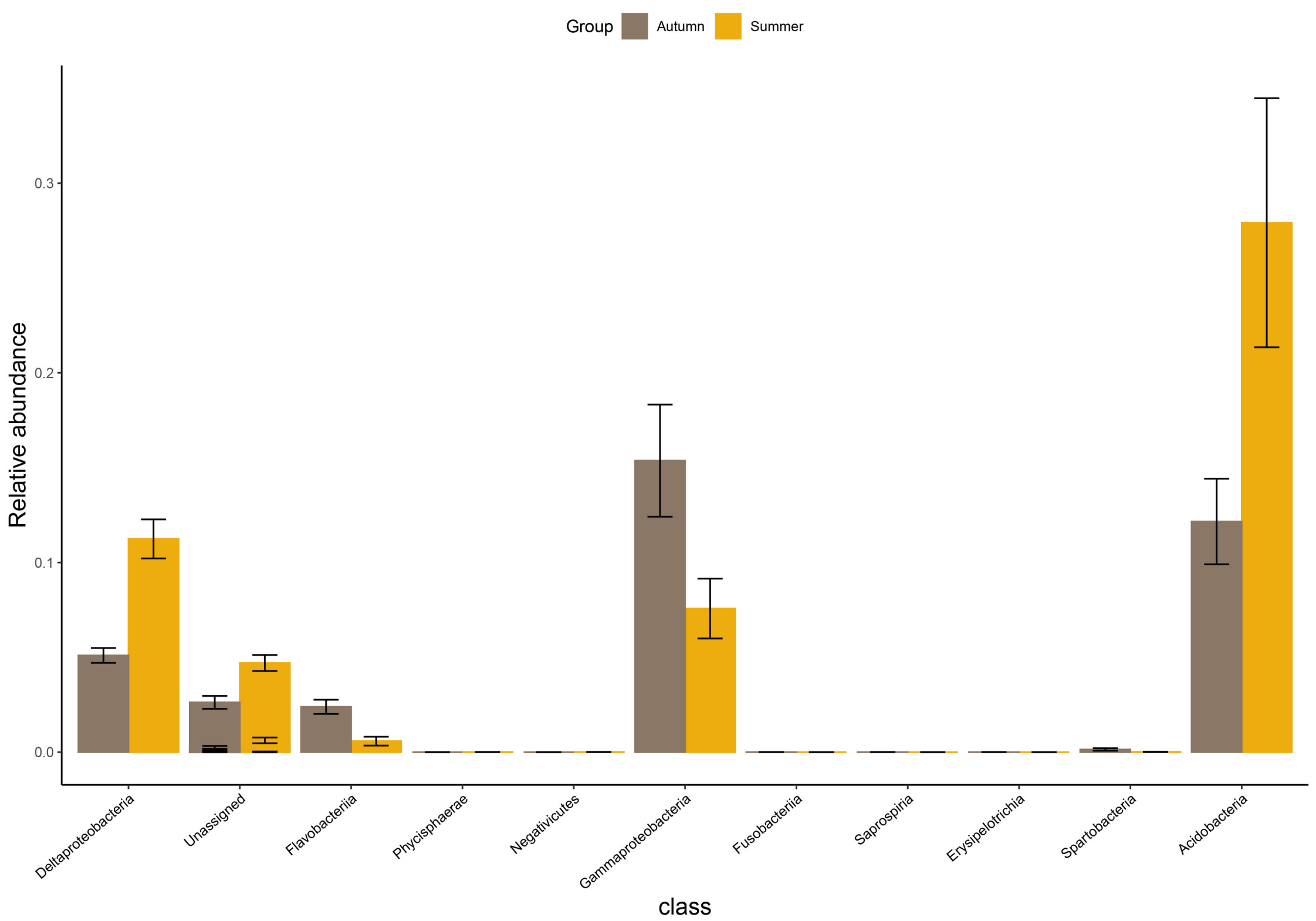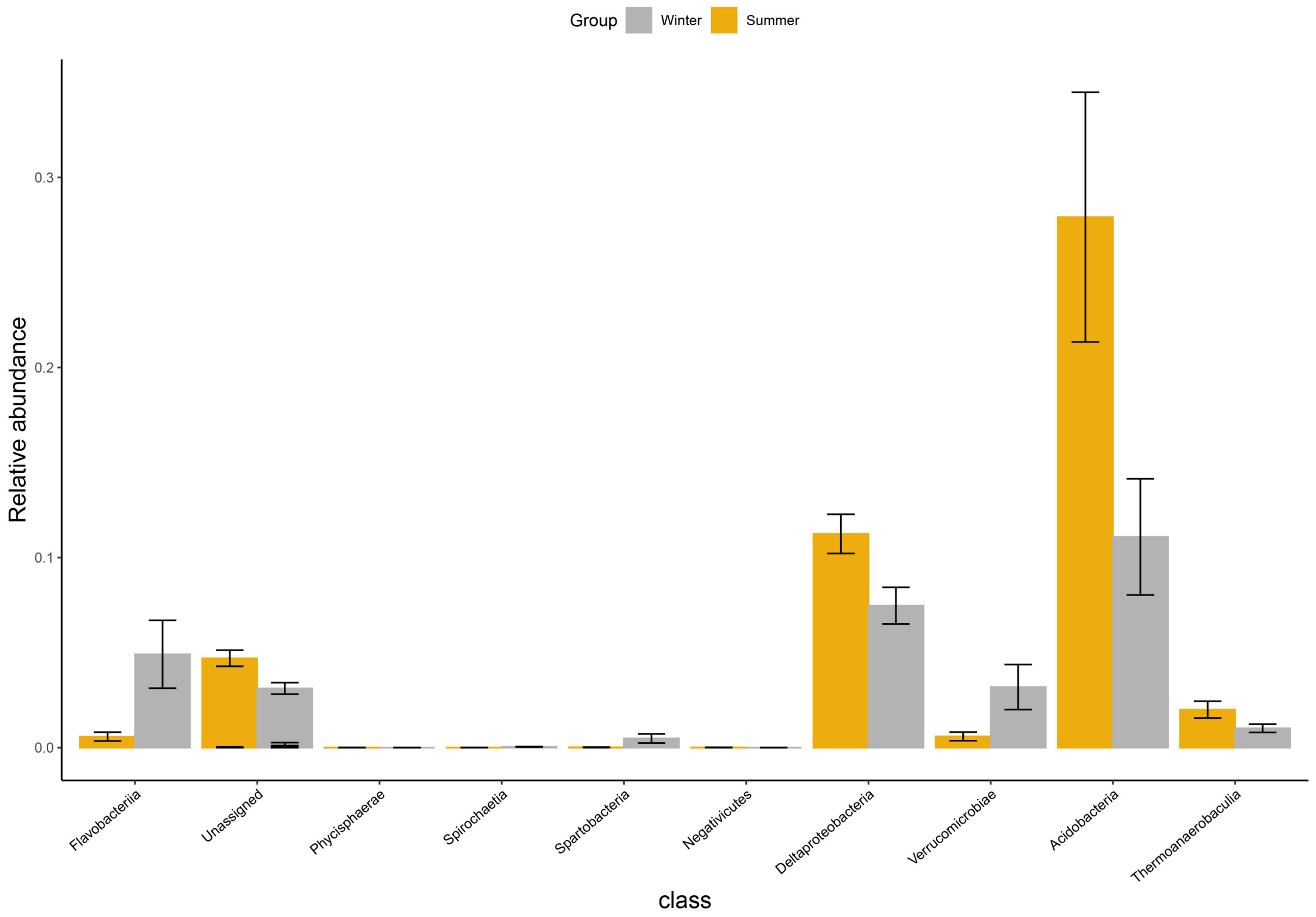

Supplement: Supplementary file 1 [file biology-11-00913-s001.zip › Figure S1. Metastat analysis showing significant differences in bacteria composition (class level) in smples from summer compared to other analyzed sea.pdf]

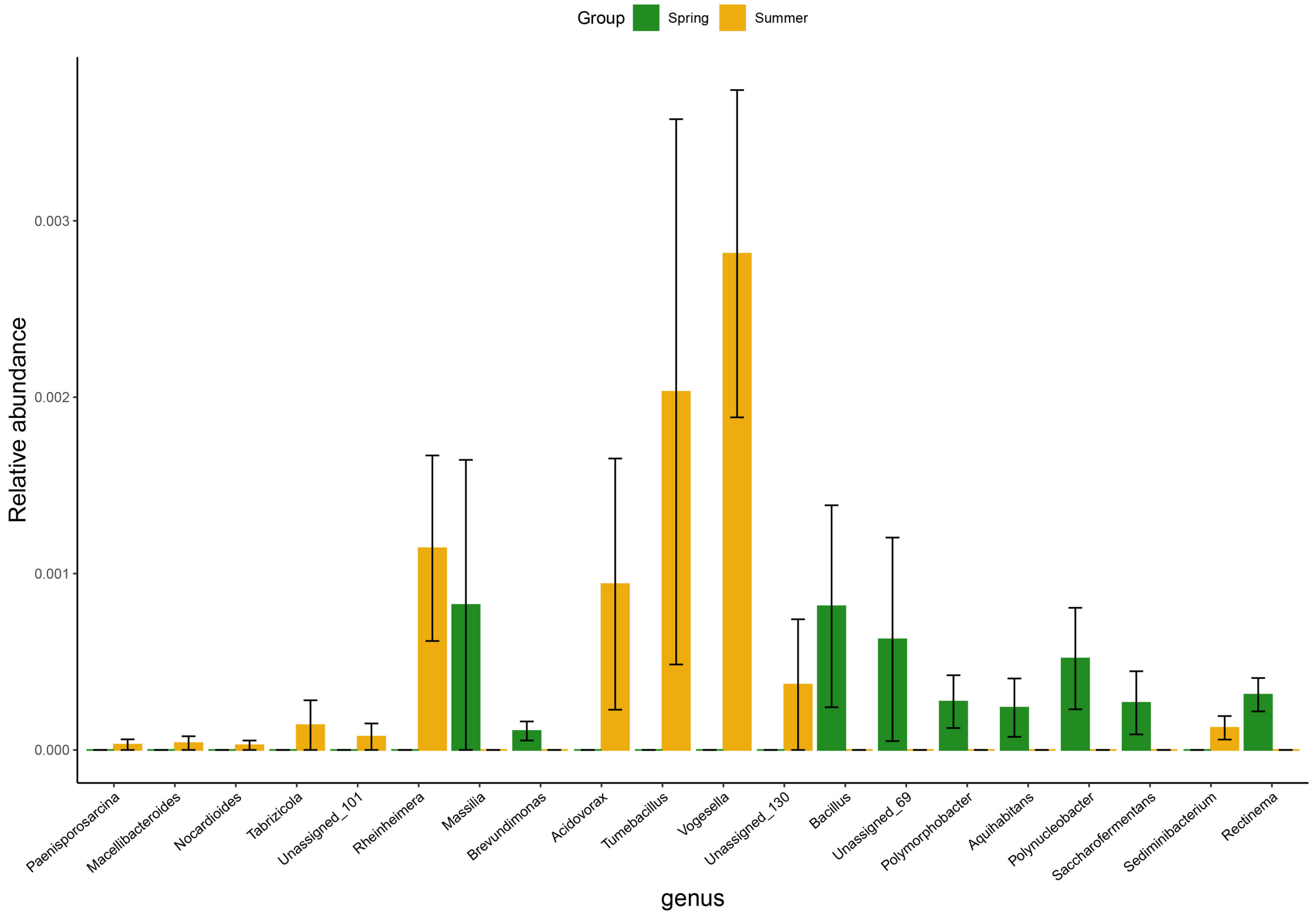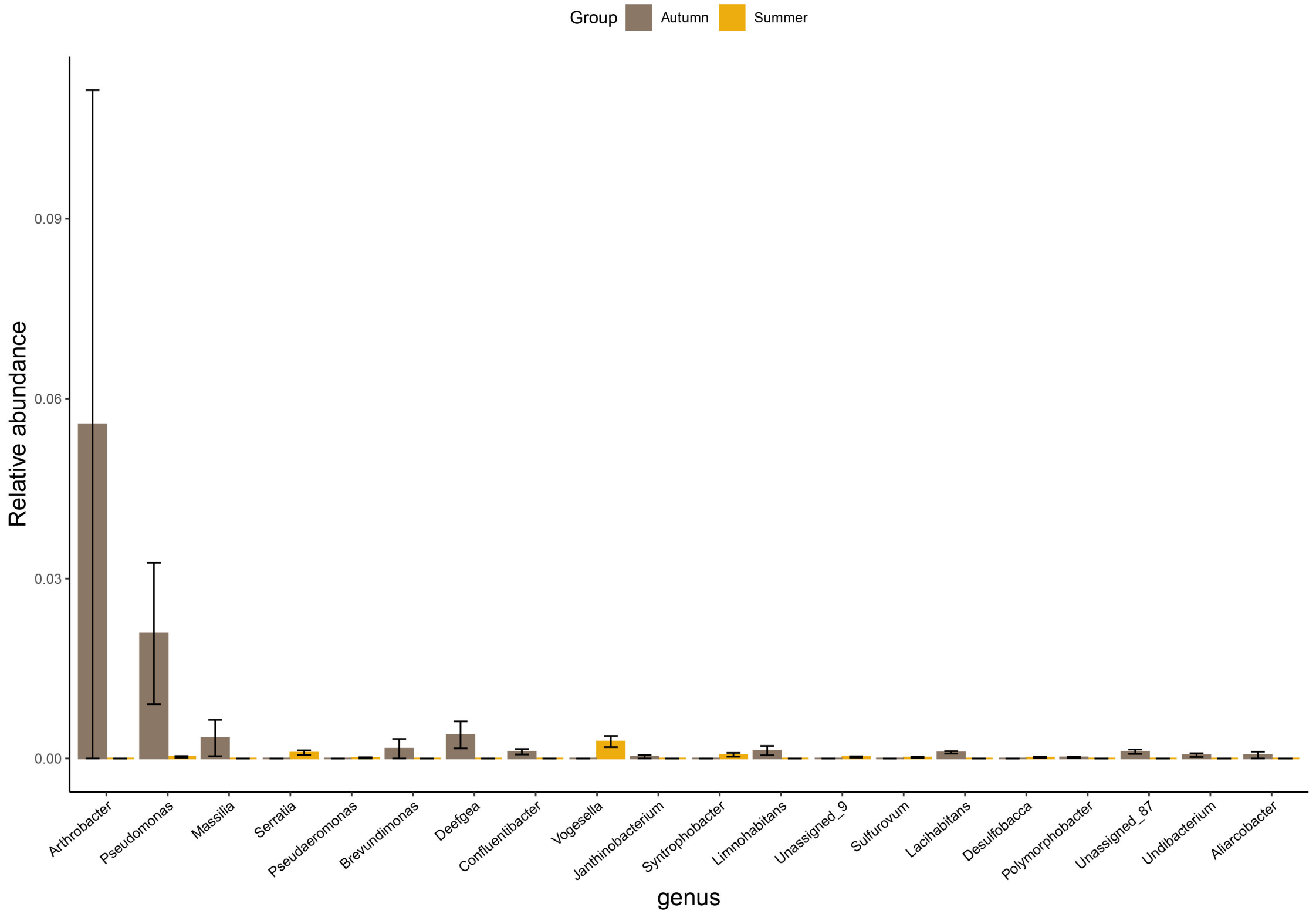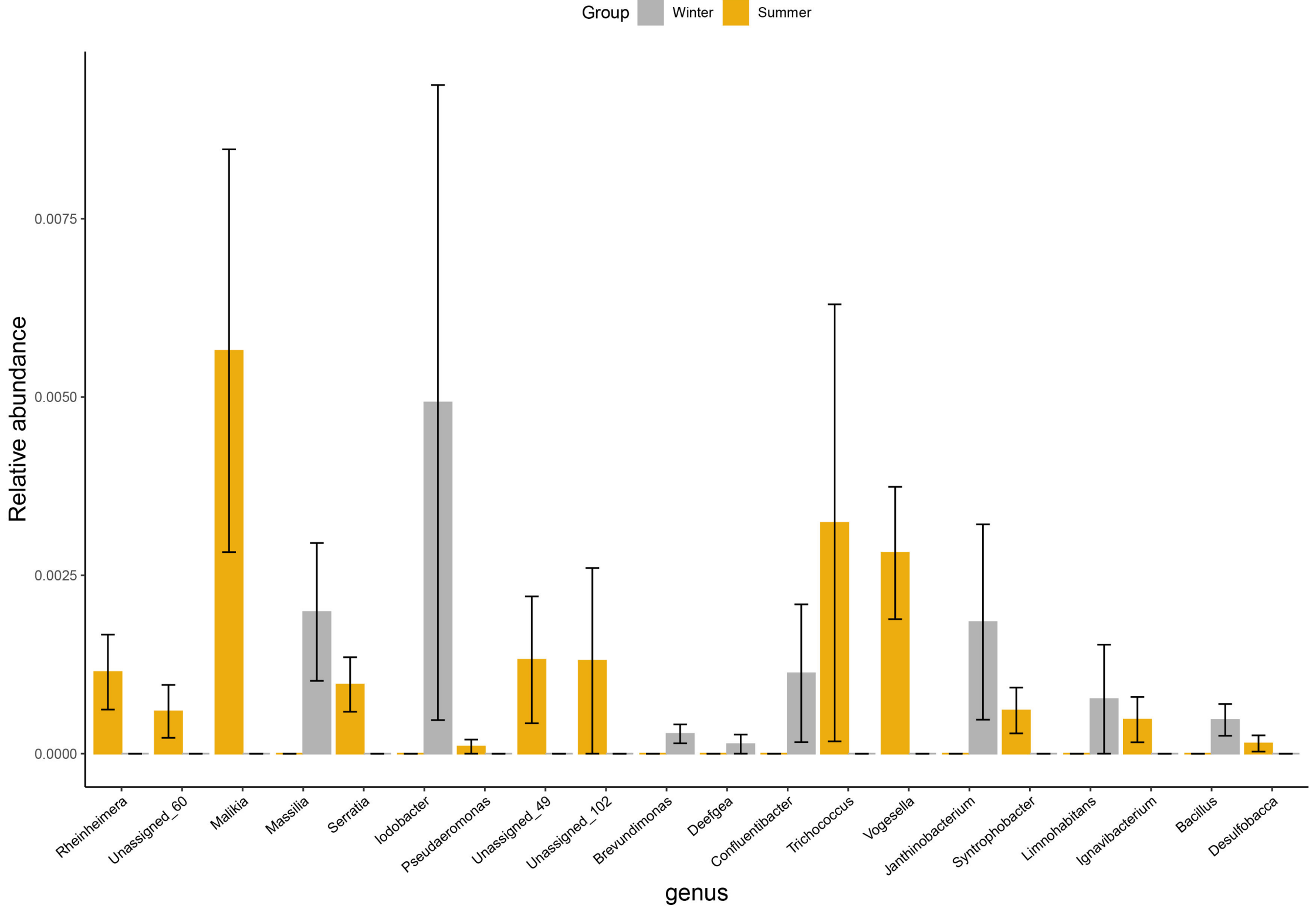

Supplement: Supplementary file 1 [file biology-11-00913-s001.zip › Figure S2. Metastat analysis showing significant differences in bacteria composition (genus level) in samples from summer compared to other analyzed sea.pdf]
